# “If you don’t stop the cycle somewhere, it just keeps going”:  Resilience in the context of structural violence and gender-based violence in rural Ontario

**DOI:** 10.1371/journal.pgph.0002775

**Published:** 2024-01-11

**Authors:** Tara Mantler, Julia Yates, Katie J. Shillington, Panagiota Tryphonopoulos, Kimberley T. Jackson

**Affiliations:** 1 School of Health Studies, Faculty of Health Sciences, The University of Western Ontario, London, Ontario, Canada; 2 Health and Rehabilitation Sciences Program, Faculty of Health Sciences, The University of Western Ontario, London, Ontario, Canada; 3 Arthur Labatt Family School of Nursing, Faculty of Health Sciences, The University of Western Ontario, London, Ontario, Canada; FLAME University, INDIA

## Abstract

Bolstering women’s resilience in the context of gender-based violence (GBV) requires attention to structural conditions needed to support women to thrive, particularly in rural communities. This cross-sectional study explored how resilience was influenced by structural violence in rural Ontario among women experiencing GBV (*n* = 14) and service providers in the GBV sector (*n* = 12). Interviews were conducted and revealed forms of structural violence that undermine resilience for women experiencing GBV in rural communities, including 1) housing- gentrification, short-term rentals of residential properties, and long waitlists, 2) income- fighting for enough money to survive, 3) safety- abusers gaming the system, and 4) access- successes and new barriers. Structural conditions must be attended to as they are prerequisites required to build resilience.

## Introduction

Gender-based violence (GBV) is rooted in gender inequality, the abuse of power, and damaging norms and can be understood as any harmful act directed at an individual based on their gender [1]. In trying to understand the perpetration of GBV, many disciplines have posited theories ranging from individual and relationship level to socio-cultural and political level explanations [2]. Regardless, the health consequences and economic cost of GBV have been well established both in Canada and globally [3–8]. Given that 1 in 3 women experience violence at some point in their lifetime, the personal, health, and economic costs position GBV as a significant public health concern and pandemic [9, 10]. One of many approaches to addressing GBV is to support women by strengthening their resilience. Within the GBV literature, resilience is defined as a dynamic process in which psychosocial and environmental factors interact to enable an individual to survive, grow and even thrive despite exposure to adversity [11]. While strengthening resilience holds promise as one way to address GBV, recent literature has focused predominantly on building individual capacity, with little attention given to the structural conditions needed to support capacity development [2].

When examining structural conditions needed to support building individual capacity, attention must be paid to both the historical and social contexts that exist and influence women. Fundamentally, our society has culturally constructed messages about gender norms and power that disadvantage women [12]. Specifically, gender norms dictate resources, roles, and power distribution according to gender, privileging those who identify as male [13–15]. Moreover, power in the context of social systems and institutions stems from social stratification and is used to influence others’ actions, beliefs, or behaviour [16]. Structural violence is one form of power exerted by social structures and/or institutions. Structural violence describes the systemic ways individuals are prevented from achieving their full potential through constraining individual agency [17, 18]. Structural violence persists when inequities become commonplace, and there is no longer outrage at how things are, signalling society-level complacency and acceptance [2]. Together, gender norms and power imbalances in society culminate in the inequities experienced by women and continue to work to reinforce these inequities once they exist [2].

Gender norms and power intersect with structural violence explaining, in part, inequitable access to the social determinants of health [2]. Inequitable access to social determinants of health such as housing, food, income, health care, and geography contributes to disparities in health [17, 19]. These health disparities are more pronounced among vulnerable populations, such as those experiencing GBV [20–22]. Understanding the intersection of structural violence, social determinants of health, and GBV, reveals the dynamics of social institutions that exist across many dimensions of people’s lives, which negatively affect resilience and undermines health [23, 24].

Rurality is an important social determinant of health that can further amplify structural violence and the consequences of GBV [25]. Research into rural GBV has focused on comparing differences to women in urban areas and has centred around the disparities in health, lack of access to health and social services, and more severe experiences of violence, including femicide [26–31]. These inequities have been attributed historically to commonly held rural community norms, such as conservative views, hegemonic masculinity, and traditional gender norms [32–36]. While these assertions are grounded in evidence, the research is largely outdated and does not account for the extensive work the GBV sector has been doing in rural communities to change these norms [37–39]. Without a current understanding of how structural violence is enacted in rural contexts among those experiencing GBV, supporting women to build resilience will remain challenging. This study aims to fill this gap by exploring how resilience is influenced by structural violence in rural Ontario in the context of GBV.

## Methods

As part of a larger qualitative study entitled “Understanding **R**ural Canadian Women who have Experienced **I**ntimate Partner Violence and the Factors that **S**hape their Resilienc**E** (RISE), the current paper serves as a sub-analysis focusing on how resilience is influenced by structural violence in rural Ontario in the context of GBV.

### Ethics statement

Ethics approval for the RISE study was obtained from the host institution (NMREB #116676). Formal consent was verbally obtained during interviews with all participants. Per the NMREB’s approval for the study, verbal consent was audio recorded and noted in the Master List by the researcher conducting the interview. All audio records for this study, including proof of consent from participants, were stored on an institutional hard drive.

### Study design

This cross-sectional qualitative study used interpretive description [40], a pragmatic approach that is both constructivist and naturalist that aims to generate knowledge for applied disciplines.

### Sampling and recruitment

Purposive and snowball sampling were used to recruit participants via rural Kijiji ads and posters in rural women’s shelters between November 2020 and June 2021. To be eligible for the larger RISE study, women needed to live in rural Ontario, have experienced IPV, and have access to a safe computer or telephone, while service providers needed to have worked at a rural women’s shelter for a minimum of six months. After recruitment, a total of 14 women and 12 shelter staff participated in this study from eight rural shelters. Women and shelter staff included in this study were from communities with populations ranging from 2,000 to 42,000 people, representing 12 different rural communities across Ontario.

### Participants

Women’s ages ranged from 18 to 57 years old (*M* = 34.86 years, *SD* = 9.31). Sixty percent of participants had received college or university-level education. In terms of employment, five women worked part-time, four worked full-time, four were unemployed, and one woman identified as self-employed. The average annual household income after taxes ranged from $15,000 to $110,000 Canadian dollars (CAD). Approximately 65% of this sample identified as heterosexual, 29% as bisexual, and 6% identified as having a fluid sexuality. The majority of women (85.71%) were of European descent, while one woman identified as Indigenous.

Most shelter staff were employed full-time (*n* = 10) with all staff having worked for their various agencies for a minimum of 6 months. Shelter staff ranged from 27 to 59 years old (*M* = 42.25, *SD* = 11.73). The education and income of the sample was generally high, with all staff achieving college diplomas and/or university degrees and half averaging an annual household income of more than $100,000 CAD (range from $32,000 CAD to $150,000 CAD).

### Procedures

Ethics approval was obtained from the host institution’s Non-Medical Research Ethics Board (NMREB #116676), and data collection occurred between November 2020 and June 2021. Individual interviews, lasting approximately 60 minutes, were conducted in two phases four months apart. Phase one interviews were conducted between November 2020 and February 2021 with women (*n* = 14) and shelter staff (*n* = 12). Phase two interviews occurred between May and June 2021, with six women and five shelter staff for the purpose of member checking and to ensure accuracy and resonance with participants’ experiences [41]. Table 1 presents the questions asked during both phases of interviews. All interviews included the researcher recording field notes and were audio-recorded and transcribed verbatim, with each transcript being anonymized prior to analysis. The data collection and analysis process were guided by Guba and Lincoln’s [41] and Thorne and colleagues’ [42] principles of auditability, fit, dependence, and transferability. To reduce barriers to participation, women and shelter staff received a $25 gift card in recognition of their time during phase 1 and a $10 gift card during phase 2.

**Table 1:** Interview questions for women and shelter staff.

Phase of Interview	Participant Group	Interview Questions
1	Women (*n* = 14)	1. What helps to support your resilience? 2. What undermines your resilience? 3. What are some challenges/barriers that you have faced to being resilient? 4. What did [do] you need to thrive over time? 5. What adaptations have you used to be resilient? 6. What has contributed [contributes] to your inner strength?
1	Shelter Staff (*n* = 12)	1. What do you think helps to support women’s resilience? 2. What do you think undermines women’s resilience? 3. What are some challenges/barriers that you have seen women encounter that prevent them from being resilient? 4. How have you seen women thrive over time? 5. What adaptations have women used to be resilient? 6. What do you think contributes to women’s inner strength?
2	Women (*n* = 6)	1. In your relationship, what made you feel stuck? How did you overcome that feeling of “stuck-ness”? 2. How would you describe your mindset, and how do you feel your mindset has played a role in your experiences, and your resilience? 3. What enabled you to keep moving on when things were difficult? When there were moments of crisis?
2	Shelter Staff (*n* = 5)	1. What forces women to stay in their relationships (or keep them “stuck” there)? How do you see women overcome that feeling of “stuck-ness”? 2. How do you feel a woman’s mindset can influence their experiences and resilience? 3. What enables women to keep moving on when things are difficult? When there were moments of crisis?

### Data analysis

Data analysis occurred after all phase on interviews were completed and then again after phase two interviews were completed. Data obtained from all interviews were organized using Quirkos qualitative analysis software [43]. Interpretive description following Thorne’s approach guided analysis [40]. The 37 transcripts were each independently coded by two of the five researchers. Initially, the principal investigator and research assistant who conducted the interviews met and created a preliminary coding structure based on field notes and what was known from the literature. Next, random coding dyads were created, and each dyad analysed two transcripts using open and line-by-line coding and provided feedback on the coding structure [44]. Subsequently the larger group met to refine the coding structure. This process was repeated two more times until the entire coding team was confident the coding structure sufficiently reflected the data. Next, all interviews were assigned to two people for final analysis. Throughout the coding process, the coding team utilized memos to identify theoretical outliers, theorize the relationship and structure of the data, and extract meaning from the data set [40]. Once all transcripts were analysed, analysis files were merged using Quirkos and queries/reports were run on codes related to structural violence as determined by group consensus on codes reflecting various aspects of structural violence. More specifically, reports were run on the following codes: *fighting for a voice*, *structural violence and oppression*, *unhelpful help*, *stigma*, *COVID-19 context*, *barriers to resilience in the rural context*, and *long-standing shelter issues*.

## Results

*“What’s hard is you would go to step forward*, *and then you would get*, *you know slapped in the face*. *And then you have to go to step back…I’d say for a good four years*, *I*, *that’s the way I was moving*, *which was hard*.*” (W [Women] 12*, *T [time*]*1)*

The findings of this research underscore forms of structural violence that undermine resilience for women experiencing GBV in rural communities, including 1) housing- gentrification, short-term rentals of residential properties, and long waitlists, 2) income- fighting for enough money to survive, 3) safety- abusers gaming the system, and 4) access- successes and new barriers. Our sample consisted of women from diverse rural communities.

### Housing-gentrification, short-term rentals of residential properties, and long waitlists

*“I think safe and affordable housing is at the root of just about every*, *everything*, *right*? *A lot of women stay in relationships*, *because they can’t get housing*, *or they*, *they stay because they can’t afford to go anywhere else*… . *So*, *I think at the end of the day*, *in order to be resilient in order to thrive*, *I think it comes down to safe*, *affordable housing*.” *(SP [Service Provider*] *12*, *T1)*

Many women experiencing violence leave their homes to be safe. This reality positions the need for safe housing as a central issue in GBV and a pre-requisite for women to build resilience. The reality that women must leave their homes because their partner abused them is the epitome of a gendered inequity. One service provider questioned and described this inequity saying, “when it comes to domestic situations, a woman has to literally uplift her whole life. Because typically the male stays in the home they shared together” (SP8, T1). With women being forced to give up their homes for safety, there is a need to ensure access to housing for women experiencing GBV. However, women and service providers both described a housing crisis in rural Ontario, specifically, a lack of access to affordable housing. While the housing crisis exists beyond rural communities, current trends in housing, such as gentrification and short-term rentals, are particularly problematic in rural communities. When gentrification occurs, houses often become unaffordable for women living in the community. One service provider detailed this trend saying, “so affordable housing, which is almost impossible to find in [municipality] now, because of all of the reasons I said before of all these people moving in here, for vacation homes, and they also do short term accommodations” (SP5, T1). A by-product of gentrification is the increase in neighbouring housing prices. This increase in value of the surrounding homes means many local women quickly become unable to afford rent in their communities. One service provider contextualized the impact of gentrification relative to income saying, “like for a room for rent in this area, you’re looking at a minimum $600 and that’s really what women are getting monthly just for income” (SP1, T1). Women not being able to afford rental prices necessitates the use of subsidized housing programs- programs which are severely overburdened in rural areas. One service provider described the dismal state of subsidized housing in her area, explaining,

A lot of times our clients will apply for rent-geared-to-income -housing which still takes a long time to get on. Typically for my rural area it’s about six months to a year with special priority status, and if they don’t qualify for special priority status, 10 years to get into other [rent geared to income] housing (SP8, T2).

The interrelated issues of gentrification, housing prices, and lack of available subsidized housing are not new, and yet what was interesting is that relatively few women experiencing GBV drew attention to this issue. When asked about housing, many women explained they were so grateful to live *anywhere* they were no longer being abused. One woman described this gratitude relative to her housing needs saying,

I could have been living in a box on the side of the road, and I would have been just happy because when you’re in a really crappy situation like that, and you- you know that you need to get out, you don’t even care where you are when you’re out (W13, T1)

### Income-fighting for enough money to survive

“But definitely, like not having income would put anybody’s resilience down, because then you don’t know what to do. And you don’t know where to go. And you don’t know to ask for help. Because it’s embarrassing” (W2, T1).

There is need for universal basic income when women leave abusive relationships. Often women leave their homes without money or sources of income, necessitating the use of social safety nets such as Ontario Works (OW) or Ontario Disability Services Program (ODSP). However, for many women interacting with OW/ODSP, these were described as a ‘fight’ or ‘struggle’ both in terms of gaining access and navigating services. One service provider described the difficultly women face when navigating OW/ODSP explaining, “it’s fighting for everything it’s fighting for, a lot of times, over half of the woman that I’ve ever dealt with have no financial income. So, it’s fighting to get it stable income in Ontario Works or ODSP” (SP8, T2). Beyond the fight for access, there is a stigma associated with using these programs that women must endure. While stigma with using these services is not uniquely a rural experience, living in a rural community means it was easier for women accessing services to be stigmatized as they were easily identified. One service provider, who also experienced GBV, described how disheartened she was with the community in terms of how women engaging with OW/ODSP were spoken about,

We’re discriminated against, you know, there’s stereotypes you know all those people who are on welfare, they’re bad, they’re bad news, or they’re losers, or low lives or, you know, there’s all kinds of labels attached to them, and you know, it’s so- it’s so sad (SP2, T1).

Ontario Works is designed to be a short-term income solution for women experiencing violence, with the intention that once women are able, they would (re)enter the workforce. However, without income, the ability to re-enter the workforce is severely hampered, as one needs to cover the cost of transport to be able to get to work. For mothers of young children, there is the added cost of procuring childcare. One service provider described the intersectional nature of income, transportation, and childcare for women explaining, “you want to get a job, you need childcare, … what’s your transportation?” (SP2, T2). Transportation difficulties are a well-established barrier to securing employment in rural communities; however, some participants described improvements in the presence of public transportation in rural communities, but the cost and unreliability of these systems undermined their utility. One service provider described the unreliability of the new transportation services, explaining “… for anybody who needs to get anything, it’s not affordable, it’s not that reliable” (SP5, T1). For women in this study, the need for universal basic income connected to their ability to build resilience as it was about having enough that they did not have to stress over basic necessities. One woman explained,

I think financial security, I think is something that is really important. It doesn’t necessarily mean any kind of element of wealth, but just rather the ability to put food on the table, put a roof over our heads, I think that that has really helped us to thrive” (W14, T1)

### Safety-abusers gaming the system

*“I find like*, *a lot of women I support are being abused through the legal system…because their partners*, *you know*, *are trying to use the legal system to get back at them…so it just drags it out goes on and on … they’re still being controlled in in different ways” (SP1*, *T1*).

Ideally, the criminal justice system, comprised of legal and policing services, should help women be safe. However, many women described negative interactions with the criminal justice system, including not being believed or, worse yet, being blamed for the violence. One woman recounted narratives she heard about herself, “it’s a lot of like victim-blaming like where it’s like ‘what did she do, she probably deserved it’ or ‘she’s a handful no wonder, I would smack her too’” (W5, T2). Being blamed for the abuse is one way the criminal justice system continues to abuse women after they have left the violent relationship, a reality that undermines resilience. One service provider explained that while gains have been made in policing services since the 1980s, abusers were continuing to learn how to ‘game’ the system,

I’ll go back, when I started in this work in the late 80s, there was the, that was the time of police being called to situations of violence in the home. And they would ask, what do you want us to do and the woman was then required to say whether or not she wanted partner charged. We then saw that everybody was getting frustrated, the courts, the police, women, women were recanting and saying no, no, no, it didn’t happen out of fear, out of pressure from their abusers, you know, many reasons. So then in the 90s, we saw where if police attendance of physical evidence of an assault having taken place, they lay the charge. That caused the abuser to change the way they abuse, no longer, sorry, less often were they leaving physical evidence, but they were using their words, and they were becoming a lot more emotionally and psychologically abusive. (SP10, T2).

Using inequities in the legal system, particularly when children were involved, was identified as a way for abusers to maintain control over women and to continue the abuse. One service provider explained the stress of going to court particularly when child protective services were involved and the reality that women are often shamed during this process, “I have heard from other services that sometimes our child protective services can undermine the resilience of women in terms of blame, shame and blame” (SP10, T1). Given the continued abuse perpetrated against women who have left abusive partners it is not surprising that using the criminal justice was stressful “so the legal system in general is pretty much a shitshow [laughter] like from the courts, to the police… and well going to court in general is just super stressful. So I try not to…” (N2, T1) and ultimately a last resort for women, “police are their last resort on their plans to keep them safe” (SP6, T1).

### Access-successes and new barriers

*“This is a systemic change that we continue to fight for as women for the women” (SP3*, *T1*)

Women discussed long-standing barriers to accessing services in rural communities such as non-existent services, “and next thing they know, their next available space is two hours away, and how do I get there?” (SP8, T1) and long waitlists, “the wait times I think add an air of hopelessness” (W5, T1). However, women and service providers both described recent substantial improvements due to the rapid shift to online service provision during the COVID-19 pandemic. One service provider described the benefits of this shift to virtual service, saying “due to the pandemic, again, the one silver lining is they can access virtual services now out of our district” (SP3, T1). Using virtual services bridged the gap of lack of services in rural communities, with one woman explaining she had access to the services she otherwise would not have in her rural community, “like in bigger city centres you have like crisis that’s 24/7 or mobile units etc.” (W5, T1). The shift to virtual services decreased wait times for services in some rural areas as well. One service provider highlighted this saying that access improved when health care appointments shifted to being done over the phone, “I have seen a change with wait times and they have, they have changed them they are a little bit quicker” (SP8, T2). Another service provider highlighted wait times for services in rural communities came more in line with those in urban centres,

I mean, you know, you go to bigger, bigger cities, and I think they face similar issues. They have waiting lists as well, so I just think it’s probably lack of the amount of services, maybe not so much that it’s rural (SP1, T1).

Despite the improvements to some long-standing barriers to service, some new barriers also emerged at the same time. For example, service providers repeatedly underscored the cost of internet as a barrier for many women in accessing virtual services. One service provider explained, “our Internet access is terrible cost, is higher yeah there are certainly some drawbacks to living rurally” (SP10, T1).

Beyond poor quality and costly internet service, another barrier identified was the service providers themselves. One woman described not being taken seriously by health care providers saying “…my injuries haven’t been taken seriously as a survivor and it’s often been chalked up to you’re a woman and it’s normal” (W6, T1). This woman described needing to advocate for basic health care, and the resultant exhaustion and impact on her mental health. Despite the clear need for health care, this woman stopped using health care as it was not worth the toll on her mental health. Service providers highlighted that for many women, reaching out to services is a significant step, and when service providers are unsupportive it negatively impacts the likelihood she will reach out again. Service providers explained that one negative or unsupportive interaction could undermine a woman’s resilience, “one negative experience I think is enough to set them back, … you know, that lack of compassion, warmth, empathy from service provider, I think that definitely has a negative impact on [the woman’s] resilience” (SP11, T1).

## Discussion

Our findings revealed how resilience is influenced by structural violence in rural Ontario communities for women experiencing GBV. While these communities are not homogenous, the purpose of this analysis was to find commonality in the rural Ontario experience. First, and consistent with other housing-related discourses, there is a housing crisis impeding women’s ability to find affordable housing in rural communities. Interestingly, while many service providers highlighted this, women experiencing GBV were so grateful for the ability to be physically and emotionally safe that concerns about housing were not mentioned. Women and service providers identified a need for universal basic income as a pre-requisite for building resilience, as women without money were unable to find transportation, childcare, or housing, thus inhibiting the ability to get a job and earn an income. The criminal justice system was identified as a barrier to building resilience, as many women reported abusers were using policing and legal services to control them and prolong the abuse. The very services that, by design, should help ensure women’s safety were services women chose not to engage with due to previous stressful and negative experiences. Traditional barriers in access to services have improved with the rapid shift to virtual services necessitated by the COVID-19 pandemic; however, the virtual delivery of services unveiled additional obstacles for women, such as the cost of the internet and negative attitudes of service providers toward those experiencing GBV.

In Canada, housing is purportedly a basic human right, yet remains the most significant longstanding challenge for women experiencing GBV [45]. There is mounting evidence that decades of divestment in social housing in Canada and the trend of financialization of housing both undermine the availability of affordable housing in Canada [46–49]. Divestment in social housing and financialization of housing results in women experiencing GBV struggling to move along the housing spectrum from shelters to permanent homes [50, 51]. Unique to this study was the impact of this housing crisis in rural areas on resilience. Service providers identified that not having a place to live was a key barrier for women in building resilience; however, women reported that their physical and emotional safety was more of a priority. Given that some women experiencing GBV are not identifying housing as a barrier, there is the potential that this pivotal environmental factor to building resilience could be overlooked. There is a need for policymakers to prioritize access to affordable housing in rural communities.

The increasing disparity between income and cost of living, exacerbated by stagnating social safety nets and rising income inequalities, presents a considerable barrier to women building resilience [52]. Social assistance programs in Ontario, such as OW and ODSP, are intended to provide temporary financial and employment assistance, including covering the cost of housing, food, and basic living expenses [53]. Yet, with rapidly rising living costs and static social assistance, these programs no longer meet individuals’ basic needs [54]. The inefficiencies of these programs, in turn, have devastating effects on recipients (e.g., vulnerability to homelessness, food insecurity, and unemployment) with marginalized populations, such as women being unequally disadvantaged [55, 56]. This inequity is further compounded when women experience GBV as basic income is needed to afford women the ability to leave abusive relationships [55–57]. Women in this study identified how the stress associated with not having access to basic income thwarted their ability to build resilience and move beyond simply surviving. Further, this study also identified that stigma in rural communities around using social assistance was a barrier to accessing needed services and further undermined women’s ability to build resilience. There is a need for policy intervention to support basic income in social safety nets and a need to reduce the stigma associated with accessing these supports.

For women to survive, grow, and even thrive, they need to be safe; however, many women in this study described negative interactions with the criminal justice system. Given how the criminal justice system is rooted in adversarial and defendant-oriented practices, it is unsurprising that women experiencing GBV report dissatisfaction with this system [58, 59]. The limited and dated research in this area is in line with the findings of this study surrounding systemic prejudices and secondary victimization [60, 61]. Despite work by the Canadian government around mandatory charges, sentencing provisions for physical and sexual violence, and a greater emphasis on how the criminal justice system responds to those experiencing GBV, improvements in this system for women experiencing GBV are slow at best [58, 62–64]. The current study’s findings echo earlier works in this area that are over 15 years old. Previous studies have underscored how interactions with the criminal justice system can be humiliating and detrimental to women’s well-being [58, 65]; a lack of police awareness about GBV often results in victim-blaming [66]; lastly, women lack control when interacting with the court system leading to re-victimization [67]. There is a need for a trauma- and violence-informed approach to be integrated into the criminal justice system to ensure women are not being further abused by systems designed to keep them safe.

The global pandemic and rapid shift to virtual services reduced historical barriers in accessing services for rural women experiencing GBV. Specifically, access to services improved and wait times decreased. However, additional barriers emerged, including internet accessibility and negative attitudes of services providers toward women experiencing GBV. The soaring cost of internet in rural areas is well-documented [68]; however, when coupled with the financial strain and insecurity women face when leaving an abusive relationship, the internet, while an essential service [69], becomes prohibitively expensive. Women’s experiences with services are primarily dictated by the service provider. For women experiencing violence, stigma and a lack of compassion and empathy were reported barriers to resilience, a lamentable finding consistent with previous research [70, 71]. Unique to this study was the impact of negative interactions with service providers on women’s resilience. This study underscored that these negative interactions meant women stopped engaging with key services that can be instrumental in building resilience. There is a need to ensure virtual services are available beyond the pandemic, that internet costs do not prohibit women experiencing violence from accessing this basic service, and that research and social services continue to address the stigma associated with GBV.

### Study limitations

This study provided a cross-sectional look at how resilience was influenced by structural violence among women who experienced GBV in rural Ontario. It is important to contextualize these findings in the context of study limitations, including recruitment and demographics. A recruitment criterion for this study was that women needed to identify as being resilient, which biased the sample. Moreover, the demographics of women and service providers in this study do not reflect the diversity of women experiencing GBV nor the Canadian population. We are missing the experiences of racialized women, women whose first language is not English, and older women. It is important that follow-up studies include women who are and are not resilient and purposive sampling to ensure adequate representation. Future studies would also benefit from gathering more in-depth data on rural communities represented in the sample so results could be further contextualized based on the attributes of the communities.

## Conclusion

In the context of rural Ontario, our study identified structural violence in housing, income, safety, and access to services as key environmental factors impacting the ability of women experiencing GBV to cultivate resilience. The lack of affordable housing, insufficient income to cover the basic cost of living, unsupportive and re-traumatizing criminal justice system, and stigma associated with GBV among service providers left women stressed, embarrassed, and not wanting to interact with services designed to support them. However, the rapid shift to virtual services during the COVID-19 pandemic meant that some historical barriers to accessing services in rural areas resolved. Policymakers need to prioritize affordable housing, basic income, trauma and violence approaches in the criminal justice system, the continued use of virtual services, and affordable internet access for rural areas to meet the needs of women experiencing GBV. When these environmental factors are in place, women will have the prerequisites required to build resilience.
